# Vaccines as alternatives to antibiotics for food producing animals. Part 2: new approaches and potential solutions

**DOI:** 10.1186/s13567-018-0561-7

**Published:** 2018-07-31

**Authors:** Karin Hoelzer, Lisa Bielke, Damer P. Blake, Eric Cox, Simon M. Cutting, Bert Devriendt, Elisabeth Erlacher-Vindel, Evy Goossens, Kemal Karaca, Stephane Lemiere, Martin Metzner, Margot Raicek, Miquel Collell Suriñach, Nora M. Wong, Cyril Gay, Filip Van Immerseel

**Affiliations:** 1 The Pew Charitable Trusts, 901 E Street NW, Washington, DC, 20004 USA; 2 Ohio Agriculture and Research Development Center, Animal Sciences, Ohio State University, 202 Gerlaugh Hall, 1680 Madison Ave., Wooster, OH 44691 USA; 3 Pathobiology and Population Sciences, Royal Veterinary College, University of London, Hawkshead Lane, North Mymms, Hertfordshire AL9 7TA UK; 4 Department of Virology, Parasitology and Immunology, Faculty of Veterinary Medicine, Ghent University, Salsiburylaan 133, 9820 Merelbeke, Belgium; 5 School of Biological Sciences, Royal Holloway University of London, Egham, Surrey TW20 0EX UK; 6 Science and New Technologies Department, World Organisation for Animal Health (OIE), 12 Rue de Prony, 75017 Paris, France; 7 Department of Pathology, Bacteriology and Avian Diseases, Faculty of Veterinary Medicine, Ghent University, Salsiburylaan 133, 9820 Merelbeke, Belgium; 8 Elanco Animal Health, 2500 Innovation Way, Greenfield, IN USA; 9 Merial, 29 Avenue Tony Garnier, 69007 Lyon, France; 10 RIPAC-LABOR GmbH, Am Mühlenberg 11, 14476 Potsdam, Germany; 11 MSD, 2 Giralda Farms, Madison, NJ USA; 12 Office of National Programs, Agricultural Research Service, USDA, Sunnyside Ave, 5601 Beltsville, MD USA

## Abstract

Vaccines and other alternative products are central to the future success of animal agriculture because they can help minimize the need for antibiotics by preventing and controlling infectious diseases in animal populations. To assess scientific advancements related to alternatives to antibiotics and provide actionable strategies to support their development, the United States Department of Agriculture, with support from the World Organisation for Animal Health, organized the second International Symposium on Alternatives to Antibiotics. It focused on six key areas: vaccines; microbial-derived products; non-nutritive phytochemicals; immune-related products; chemicals, enzymes, and innovative drugs; and regulatory pathways to enable the development and licensure of alternatives to antibiotics. This article, the second part in a two-part series, highlights new approaches and potential solutions for the development of vaccines as alternatives to antibiotics in food producing animals; opportunities, challenges and needs for the development of such vaccines are discussed in the first part of this series. As discussed in part 1 of this manuscript, many current vaccines fall short of ideal vaccines in one or more respects. Promising breakthroughs to overcome these limitations include new biotechnology techniques, new oral vaccine approaches, novel adjuvants, new delivery strategies based on bacterial spores, and live recombinant vectors; they also include new vaccination strategies in-ovo, and strategies that simultaneously protect against multiple pathogens. However, translating this research into commercial vaccines that effectively reduce the need for antibiotics will require close collaboration among stakeholders, for instance through public–private partnerships. Targeted research and development investments and concerted efforts by all affected are needed to realize the potential of vaccines to improve animal health, safeguard agricultural productivity, and reduce antibiotic consumption and resulting resistance risks.

## Introduction

Alternatives to antibiotics can help minimize the need for antibiotics by helping to prevent and control infectious diseases in animal populations. As such, safe and effective alternatives are crucially important to the future success of animal health and production. To assess scientific advancements in the research and development of alternatives to antibiotics, highlight promising research results and novel technologies, assess challenges associated with their commercialization and use, and provide actionable strategies to support their development, the United States Department of Agriculture (USDA), with support from the World Organisation for Animal Health (OIE), organized the second International Symposium on Alternatives to Antibiotics. The symposium focused on six key areas: vaccines; microbial-derived products; non-nutritive phytochemicals; immune-related products; chemicals, enzymes, and innovative drugs; and regulatory pathways to enable the licensure and development of alternatives to antibiotics. This two-part manuscript synthesizes and expands on the scientific presentations and expert panel discussions from the symposium regarding the use of vaccines as alternatives to antibiotics that can reduce the need for antibiotic use in animals. Part 1 synthesizes and expands on the expert panel discussions regarding the opportunities, challenges and needs related to vaccines that may reduce the requirement for use of antibiotics in animals, while part two focuses on highlighting new approaches and potential solutions.

A general discussion of the importance of antibiotic resistance and the opportunities, challenges and needs related to vaccines as alternatives that may reduce the need for use of antibiotics in animals is provided in part 1 of this review, including a discussion of the properties of ideal vaccines, how current vaccines compare to these ideal vaccines, and how investment decisions around research and development of vaccines are made. This second part of the manuscript will highlight specific research advancements in the area of veterinary vaccines.

## New approaches for the development of veterinary vaccines

### Mucosal immunity and tolerance: challenges to the development of effective oral vaccines

As mentioned in part one of this manuscript, most pathogens invade the host at the mucosal surfaces, such as the gastro-intestinal (GI) tract. The GI tract constitutes the largest surface area of the body and is exposed daily to vast numbers of foreign antigens derived from feed, the microbiota and pathogens [1]. Within the intestine a complex cellular network has evolved to prevent unwanted immune responses to innocuous antigens, for instance feed or microbiota, while allowing swift protective responses against agents that cause infectious disease. Key to keeping enteric pathogens at bay is the presence of protective pathogen-specific secretory IgA (SIgA) at the site of entry, which prevents the adhesion of micro-organisms to the intestinal surfaces and neutralizes their enterotoxins. Triggering robust and protective intestinal SIgA responses usually requires the local administration of vaccines [2]. Although live attenuated oral vaccines have had tremendous success, resulting for instance in the near global eradication of poliovirus [3], concerns on the dissemination of vaccine strains into the environment and rare cases of reversion to virulence, leading to vaccine-induced disease, have driven oral vaccine development to nonliving or vectored vaccines [4]. However, oral vaccination is challenging due to several hurdles imposed by the cellular and molecular architecture of the gut: (i) the harsh environment of the stomach and small intestine, including the low pH, digestive enzymes, and bile salts, required to digest feed also easily destroys vaccines, (ii) a poor uptake of vaccine antigens by the intestinal epithelial barrier and (iii) the tolerogenic mechanisms that pervade the intestinal tissues, leading to peripheral and oral immune tolerance upon oral administration of antigens via the induction of FoxP3^+^ regulatory T cells. This often results in a low immunogenicity of oral vaccines and requires innovative strategies to deliver the vaccine antigens to the intestinal immune system as well as the inclusion of adjuvants that promote innate and adaptive immunity [5].

The mucosal immune system in the gut can be divided in inductive sites, where sampled antigens stimulate naive T and B cells, and effector sites, where effector cells perform their functions, e.g. assisting in the production of SIgA. In the small intestine, the inductive sites comprise the gut-associated lymphoid tissues (GALT) and the mesenteric lymph nodes, while the effector sites constitute the lamina propria and the surface epithelium [6]. The GALT itself is composed of Peyer’s patches (PP), appendix and isolated lymphoid follicles. The presence of other GALT-like structures, such as lymphocyte-filled villi (rat, human) and cryptopatches (mouse) is dependent on the species. Interestingly, while in birds and most mammals PP or their equivalent are scattered throughout the small intestine, in pigs, ruminants and dogs the PP in the distal small intestine (ileum) are continuous. Fish and reptiles on the other hand lack PP and the intestinal immune system in these species is composed of epithelial leukocytes and rare, small non-organized lymphoid aggregates. It remains largely unknown how these species-specific differences might affect the efficacy of oral vaccines.

From their entry point, which is typically the oral cavity, to their delivery site, most commonly the small intestine, the integrity of delivery systems and the stability of vaccine components are at risk. Lysozyme in saliva, the low gastric pH together with pepsin and intestinal proteases can degrade oral vaccines. Enteric coating of vaccine components with pH-responsive polymers with a dissolution threshold of pH 6 might protect against gastric degradation and results in the release of their contents in the small intestine [7]. In this context, ruminants pose an additional problem to vaccine stability as their polygastric gastro-intestinal tract effectively degrades substances including vaccines. Site-specific delivery of oral vaccines to the small intestine is favorable as the mucus layer covering the small intestinal epithelium consists of only one layer, which is loosely adherent, less thick and patchy as compared to the colonic mucus layers and might promote their access to the intestinal epithelium. In addition, the small intestine is less densely populated by the microbiota, which might further disrupt the integrity of the delivery systems and the stability of vaccine components. Underneath the mucus layer, a single layer of intestinal epithelial cells prevents uncontrolled access of the luminal content to the underlying intestinal tissues, further restricting uptake of oral vaccine antigens. Crossing of the epithelial barrier by vaccines could be enhanced by exploiting antigen sampling routes in the small intestine or by adopting strategies used by enteric pathogens to colonize or invade the host [8]. The best-known sampling route in the gut is associated with microfold (M) cells. These specialized intestinal epithelial cells reside within the follicle-associated epithelium covering the Peyer’s patches and take up macromolecules, particulate matter and microorganisms [9]. Many enteric pathogens hijack M cells to invade the host by binding to apical receptors. For instance, the invasin protein of *Yersinia* species interacts with β1 integrin on M cells, leading to infection [10]. Likewise, GP2 marks M cells in many species and binds to FimH, a subunit of type I pili on *Escherichia coli* and *Salmonella enterica*. This interaction results in uptake of FimH^+^ bacteria and initiates mucosal immunity [11]. Although many groups have focused on improving antigen uptake by targeting oral vaccines to M cell-specific receptors, these cells represent only a small, species-specific percentage of the total intestinal epithelial cell population. Although M cell numbers increase from the cranial to caudal small intestine and M cell targeting strategies work quite well in rodent models, they mostly fail in larger animals due to the long passage time needed to reach the distal small intestine, where the gut-associated immune system is most pronounced. Besides M cells, sampling of luminal antigens also occurs by intestinal mononuclear phagocytes via transepithelial dendrites. This sampling mainly occurs by CD11c^+^CX3CR1^+^ macrophages, which transfer the antigens to CD103^+^ dendritic cells (DCs). These DCs then drive the differentiation of regulatory T cells (Tregs), which subsequently induce tolerance to these proteins [12]. In the steady state, goblet cells can also transport small soluble proteins (<10 kDa) across the epithelium to tolerogenic DCs via so-called goblet cell-associated antigen passages [13]. Absorptive intestinal epithelial cells or enterocytes, constituting >90% of the small intestinal epithelium, may also sample the luminal content through receptor-mediated transcytosis. For instance, the neonatal Fc receptor (FcRn), an MHC class I-like Fcγ receptor, is expressed on the apical surface of enterocytes and transcytoses IgG, immune complexes or Fc-coated nanoparticles from the lumen to the basolateral surface of the epithelium [14]. Similar to M cells, it might be worthwhile to target apical receptors exploited by enteropathogens on small intestinal enterocytes to promote uptake of antigens by the epithelial barrier. A potential candidate would be aminopeptidase N (ANPEP), a zinc-dependent peptidase present in the brush border of small intestinal enterocytes, which serves as an entry receptor for several coronaviruses and also binds F4 fimbriae, a colonisation factor produced by porcine-specific enterotoxigenic *E. coli*. ANPEP also transports F4 fimbriae as well as microparticles functionalised with ANPEP-specific monoclonal antibodies across the intestinal epithelial barrier, resulting in robust intestinal SIgA responses, at least in piglets [15, 16].

Although the selective targeting of vaccine antigens to apical receptors might promote their uptake by the epithelium via transcytosis, this process is in itself insufficient to trigger protective intestinal immunity upon oral vaccination and explains the need to include adjuvants. These adjuvants should act on antigen presenting cells as well as intestinal epithelial cells to promote the induction of protective SIgA and cell-mediated immune responses. Indeed, enterocytes not only provide a physical barrier separating the intestinal lumen from the host tissues, but also relay information on the luminal content to the underlying immune cells through the secretion of inflammatory or tolerogenic mediators. For instance, during the steady state, enterocytes produce thymic stromal lymphopoëtin (TSLP) and transforming growth factor (TGFβ), which imprint a tolerogenic phenotype on intestinal dendritic cells [17]. In contrast, upon infection enterocytes secrete IL-6 and IL-8 [18]. This probably facilitates a switch from a tolerogenic to an immune-inductive environment, allowing activation of intestinal antigen presenting cells. As yet the most effective adjuvants for oral application are the enterotoxins from *Vibrio cholera* (CT) and enterotoxigenic *E. coli* (ETEC) (LT). Due to inherent toxicity, dmLT was developed, a nontoxic LT mutant retaining its adjuvanticity. This dmLT triggered intestinal memory responses upon oral vaccination with a nonliving ETEC vaccine and seems a promising candidate to be included as adjuvant in oral vaccines [19, 20]. Similarly promising strategies have been reported for *Eimeria* [21]. Recent studies have shown that *Eimeria*-induced IL-17 production is critical in the initiation of early innate immune response in coccidiosis and blocking of IL-17 production by exogenous IL-17-neutralizing antibody reduced both the intracellular development of *Eimeria* and the severity of intestinal lesion [22–24].

In summarizing this part, future design of oral vaccines should be tailored to the needs of the target species, focus on the selective delivery of vaccines to epithelial receptors to promote their transport across the epithelial barrier, induce protective immune response in the target tissues, and should include a mucosal adjuvant able to trigger memory SIgA responses.

### Recombinant *Bacillus* spores as oral vectored vaccines

Endospores, or spores, are produced by many bacteria as a response to nutrient deprivation. The spore is a dormant entity about 1 μm in size that can germinate, allowing a nascent cell to emerge and enter vegetative cell growth [25]. The spore carries remarkable resistance properties, being typically resistant to high temperatures (typically 70–80 °C), desiccation, irradiation, and exposure to noxious chemicals [26]. The two principal spore-forming bacterial genera are *Bacillus* and *Clostridia* with the latter being exclusively anaerobic.

Members of the *Bacillus* genus are being used as probiotics, that is, microorganisms that are added to the diet to improve the balance of microbial communities in the GI-tract and are therefore beneficial to human or animal health [27, 28]. Typical species include *Bacillus clausii*, *Bacillus coagulans* and *Bacillus subtilis*. For a long time, it has been assumed that *Bacillus* spores are soil organisms yet the evidence supporting this is actually rather sparse. Instead, spores are found in the soil in abundance but live, vegetative cells, are rarely if ever found other than in association with plants or in the animal gut. Mounting evidence shows that spores, although found in the soil, are mostly dormant and are shed in the feces of animals, which are their natural hosts [29]. The consumption of spores associated with soil-contaminated plant matter enables spores to enter the GI-tract, transit the gastric barrier unscathed and then germinate and proliferate in the intestine before excretion as dormant spores [30]. Evidence suggests that spore forming bacteria comprise as much as 30% of the gut microbiota, indicating that the ability to form spores enables bacteria to survive in the environment as well as entering and transiting the gastric barrier of animals [31].

The extraordinary resistance properties of *Bacillus* spores coupled with their ease of genetic manipulation, and their successful use as probiotics, makes them attractive candidates for the delivery of heterologous antigens for vaccination. Spores have been used as vaccine vehicles in a number of ways, differing principally in whether spores are genetically modified or not. In all cases *B. subtilis* has been utilized due to the excellent genetics available. Using genetic modification, a chimeric gene consisting of a fusion between a *B. subtilis* anchor gene and an open reading frame encoding a putative protective antigen is first constructed. The next step is introduction of the chimera into the *B. subtilis* chromosome using a gene transfer technique, typically DNA-mediated transformation, a process in *B. subtilis* that is straightforward. Typically, the anchor is the 5′-end of a gene encoding a spore coat protein such that the chimera is displayed on the spore coat. Surprisingly, heterologous antigens displayed on *B. subtilis* spores are mostly stable and do not appear to suffer extensive degradation. Using this approach a number of candidate antigens have been displayed and then evaluated in animal models. For example, spores displaying a tetanus antigen TTFC conferred protection to a lethal dose of tetanus toxin when administered orally [32, 33]. Mice dosed orally with spores expressing part of the alpha toxin of *Clostridium perfringens* were protected to challenge with alpha toxin [34]. A more recent example is that of *Clostridium difficile* where a C-terminal fragment of the toxin A (TcdA) could be stably expressed and when administered orally to hamsters conferred protection to *C. difficile* infection [35, 36]. This particular vaccine has now entered clinical evaluation in humans [37].

Using a non-genetically modified organism (GMO) approach it has been shown that spores can adsorb antigens efficiently onto their surface and surprisingly this is both strong and stable, and reflects the unique biophysical properties of the spore [38]. For the adsorption approach, it has been shown that the gastric barrier is particularly corrosive and adsorbed antigens are labile, but for intranasal delivery this method appears satisfactory. Using this approach inactive (killed) spores can be used and success has included studies showing protection to influenza (H5N1) [39] and significant reduction in lung counts of animals challenged with *Mycobacterium tuberculosis* [40]. A unique feature of spores is their ability to enhance immune responses and this adjuvant effect has been characterized in depth [41–43].

However, the use of spores as mass-delivery vehicles for vaccines has several limitations. Oral delivery clearly is the preferred approach but appears to work effectively only for the GMO approach. Oral delivery also raises issues of tolerance and may prove to be a limiting factor. Sublingual delivery has also been explored; this approach appears to provide levels of protection that are equivalent to oral delivery, but requires more doses [36, 44]. Nasal delivery is suitable but raises potential safety issues. For animal vaccines, spores are attractive since they are currently used as feed probiotics but also because they can survive the high temperatures used for feed production and may offer long-term utility. As mentioned already, spores have been manipulated for protection against *C. perfringens* but there now exists the opportunity to develop spores for protective vaccination to necrotic enteritis, an important poultry disease caused by *C. perfringens* that has been identified as a high vaccine research priority by the OIE *ad hoc* Group (see Additional file 2 in http://doi.org/10.1186/s13567-018-0560-8).

One application that is particularly promising is the use of spore vaccines in aquaculture. With intensive fish farming, *Bacillus* spores are being used as probiotic feed supplements. For shrimp farming, viral diseases have devastated the industry and one of the most important shrimp pathogens is white spot syndrome virus (WSSV) that causes seasonal outbreaks of disease [45]. A number of groups have developed *B. subtilis* spores that display the VP28 capsid protein of WSSV and when administered in feed appears to protect against white spot disease [46–49]. The mechanism for protection is intriguing; even though shrimp are not thought to produce antibodies, it is clear that presentation of the viral antigens does produce some level of specific immunity.

Despite the progress being made with spore vaccines one key issue remains: the containment of GMOs. Because spores are dormant with the potential to survive indefinitely in the environment, the use of recombinant spores in spore vaccines is likely to raise environmental concerns and successful regulatory approvals may be slow or impossible to secure. For human use, it is likely that a case can be made that the recombinant spore vaccines addresses an unmet clinical need, but for animal use devising a method for biological containment will be crucial.

### Genetically modified live microorganisms as oral vectored vaccines and vaccine platforms

Technological advancements now make it possible to genetically engineer bacteria and other microorganisms that deliver heterologous antigens in a way that can stimulate mucosal as well as humoral and cellular systemic immunity [50]. Multiple species of bacteria including *Salmonella* Typhimurium, *Salmonella* Enteritidis, *Salmonella* Typhi, *E. coli*, *Lactococcus lactis*, *Lactobacillus casei*, *Lactobacillus reuteri, Bacillus subtilis*, and *Bacillus thuringiensis*, have been used to express protein antigens derived from bacterial, viral, and protozoal pathogens [51–61]. Some of these vectors are inherently non-pathogenic; *Lactobacillus* and *Lactococcus* strains, for instance, are “Generally Recognized as Safe” (GRAS) [50, 61]. In other cases the microorganisms have been rendered non-pathogenic through the targeted deletion of virulence genes; strategies for the development of *Salmonella* vectors, for instance, typically rely on the deletion of certain metabolic functions that limit the bacterium’s ability to replicate in the host and attenuate virulence without impacting host colonization or invasion [50]. In fact, an intrinsic property shared by many, although not all, microorganisms used as vectors is their ability to effectively infect the host and initiate innate and subsequent adaptive immune responses, for instance by triggering the host’s pattern recognition receptors [50]. These recombinant vectored vaccines can be delivered directly to a mucosal surface via nasal, ocular, or oral administration, which not only allows for mass application but may also enhance mucosal immune responses, the primary surface through which most pathogens invade. Moreover, contrary to traditional attenuated live vaccines, these recombinant vaccines in many cases do not carry a risk of reversion [50].

In veterinary medicine, oral vectored vaccines have been instrumental in the eradication or control of rabies in wildlife reservoirs [62, 63]. Oral vectored vaccines have also been developed for several other veterinary applications, including some economically important diseases of food-producing animals that are associated with considerable antibiotic use such as porcine circovirus type-2 (PCV-2); in some cases, the vaccine vector is a chimera containing parts of multiple microorganisms—for instance, an attenuated live vaccine may be used as the vector—and the resulting vaccine simultaneously confers protection against multiple diseases, for instance Marek’s disease and infectious bursal disease or Newcastle disease and avian influenza [63, 64].

The development of some vaccine vector systems has been very successful and numerous veterinary vaccines have been developed based on them; the canarypox virus vector system ALVAC, for instance, has been used for the development of a range of veterinary vaccines including against rabies, influenza, and West Nile virus [64]. Similarly, adenovirus vectors have also been widely used in veterinary medicine, both in companion and food-producing animals [65]. Vaccine platforms such as these are particularly valuable as they can allow for the rapid development of vaccine candidates in response to emerging vaccine needs, but the possibility of anti-vector immunity can restrict their usefulness [66]. Research and development of additional vaccine vector platforms is therefore needed. *Salmonella* strains that express foreign antigens, either chromosomally or plasmid-based, have yielded promising results in several species including mice, humans, pigs and chicken [67–72]. Diseases for which these *Salmonella* vectored vaccines were investigated include influenza, *Brucella abortus*, post-weaning diarrhea and heterologous strains of *Salmonella* [69–72]. The use of *Pasteurellaceae* as vectors for modified live vaccines against shipping fever in calves is currently under investigation, with promising preliminary findings [73]. Use of this vector system for other diseases including pinkeye has been suggested [73].

### New approaches for in-ovo vaccines

In-ovo vaccination is a mass-vaccination strategy that is mainly used in broiler chickens albeit occasionally also in broiler-breeder and layer chickens [74]. Eggs are injected in the hatchery, typically during the third week of embryonic development around day 18 or 19. To vaccinate, a small hole is made in the shell at the blunt end of the egg and the vaccine is injected below the chorion-allantoic membrane into the amniotic cavity or directly into the embryo. Commercial in-ovo vaccination systems that automatically inject the eggs have been available since the early 1990s. More than 90% of broiler chickens in the US are vaccinated in ovo, and in Brazil that fraction equals 70% [75]. The most common use of in-ovo vaccination is for Marek’s disease, potentially combined with vaccines against other diseases such as Gumboro or Newcastle disease.

The ability to deliver a clearly defined vaccine dosage to every single chick and to invoke early protection in the chicks is among the main benefits of this technology, but it is labor-intensive, causes stress for the chicks, and high sanitary standards need to be followed during vaccine preparation and injection to manage infection risks [74, 76]. In addition, the location of the vaccine injection is critical for efficacy. It has been shown, for instance, that if Marek’s disease vaccine is accidentally deposited into the air cell or allantoic fluid, adequate protection is not achieved [77]. The stage of embryonic development can have profound effects on vaccine safety and efficacy [78]. One study, reported that vaccination of 10–12 day-old embryos with herpes virus of turkeys (HVT) led to pronounced lesions and embryonic deaths, while vaccination on days 16 did not cause detectable lesions [78]. Embryonic age at vaccination has also been shown to be correlated with antibody titers [79]. Maternal antibody titers actually increase after the typical age for in-ovo vaccinations and peak just after hatch [76]. This can interfere with proper vaccine responses. However, evidence suggests that some vaccine strains are more affected by maternal antibodies than others [80]. Deliberate vaccine development may therefore limit the often disruptive effects that can be caused by maternal antibodies [78]. Other factors that need to be considered in the development of a successful in-ovo vaccination program include the characteristics of the vaccine or vaccines to be used, the type of incubator in which the eggs are housed in the hatchery, and the breed and age of the parent flock [76].

In-ovo vaccination strategies are promising means of reducing antibiotic use in poultry production and have been the subject of intense research. Importantly, they can provide robust and early protection against immune suppressive diseases such as infectious bursal disease [81, 82] and vaccines against multiple diseases have been successfully combined. For instance, studies have shown that in-ovo vaccination strategies can simultaneously confer protective immunity against Marek’s disease, infectious bursal disease, Newcastle disease, fowl poxvirus, coccidiosis, and necrotic enteritis [83, 84]. Other combination vaccines under investigation include vectored vaccines that simultaneously provide protection against Newcastle disease and infectious bursal disease [85]. In-ovo vaccination strategies have also been explored for other poultry diseases with promising results. This included an avian influenza vaccine based on a non-replicating human adenovirus vector [86], a recombinant viral vector vaccine against infectious laryngotracheitis [87], recombinant protein *Eimeria* vaccines [84, 88, 89] and a fowl adenovirus vectored vaccine against inclusion body hepatitis [90], among many others. A *Mycoplasma gallisepticum* vaccine for in-ovo vaccination of layer chickens has also recently been evaluated, even though high chick losses at hatch were reported for the medium and high doses of the vaccine that were investigated [91]. Therefore, in-ovo vaccination strategies are capable of controlling several economically important poultry diseases. Many of these diseases are viral, but can predispose animals to secondary bacterial infections. Therefore, in many cases, in-ovo vaccines are promising alternative approaches to the use of antibiotics.

## Vaccination strategies to reduce antibiotic use for diseases from ubiquitous pathogens

### Towards the development of new *Clostridium perfringens* vaccines

*Clostridium perfringens* is widespread in the environment and in the gastrointestinal tract of most mammals and birds. However, this bacterium is also one of the most common pathogens of food-producing animals, causing disease only under circumstances that create an environment which favors growth and toxin production, such as stress, injury, or dietary changes [92]. The bacterium itself is not invasive, but causes disease through the production of a wide array of toxins and enzymes. However, no single strain produces this entire toxin repertoire, resulting in considerable variation in the toxin profiles and disease syndromes produced by different toxinotypes of this bacterium [93]. While some of these toxins act only locally, other toxins which are produced in the gut exert their action in other internal organs or can act both locally and systemically [94–96]. To date, efficacious vaccines are only available for the diseases caused by systemic action of the toxins and vaccination against enteric diseases still remains a challenge. However, some of these enteric diseases caused by *C. perfringens* are of major economic importance and lead to considerable use of antibiotics. Amongst them are necrotic enteritis in broilers and necro-haemorrhagic enteritis in calves. Despite the fact that much research is being directed to the development of novel vaccines against these *C. perfringens*-induced enteric diseases, several key barriers still have to be overcome.

In general, clostridial vaccines require multiple doses to achieve full immunity. Unfortunately, parenteral booster immunizations are impossible in the broiler industry, where mass parenteral vaccination is only feasible at the hatchery, either in ovo or on day-old chicks. Because single parenteral vaccination at day of hatch offers no protection, other delivery methods need to be developed [97]. Oral vaccines can more easily be administered to birds, without the need of individual handling of the chicks and are therefore recommended. However, some questions arise when developing an oral vaccine as compared to the parenteral administration route. In addition to the fact that maternal antibodies can block the immune response in young chicks, also the induction of oral tolerance has to be circumvented and an efficient way to present the antigens to the mucosal immune system has to be developed. Oral tolerance is a common problem in mammals and fish when developing oral vaccines. This is in contrast to chickens, where oral tolerance is age-dependent, and only an issue in 1- to 3-day-old chicks. After that age, protein antigens have been shown to induce a robust immune response and oral vaccination schemes are thought to be feasible [98]. One appealing strategy for the delivery of vaccine candidates to the mucosal immune system is the use of attenuated or avirulent bacteria as antigen vehicles [99]. Attenuated recombinant *Salmonella* strains which express *C. perfringens* antigens have been tested in several studies as oral vaccine vectors, leading to some promising results. However, the amount of protection afforded by these vaccines is not as high as compared to multiple doses of parenteral vaccination, and seems to depend on the colonization level and persistence of the vaccine strain [100–103]. This indicates that the use of live vectors to express antigens derived from *C. perfringens* strains in the gut of broilers is a promising approach, but the vaccine delivery strategy still needs to be optimized to achieve optimal antigen presentation to the mucosal immune system and provide improved protection. Alternatives to attenuated *Salmonella* strains can be *Bacillus subtilis* spores or *Lactobacillus casei*, which both have a GRAS status and have the potential to be used as vaccine carriers for *Clostridium* antigens [34, 104]. *B. subtilis* has the advantage that the heat-stable spores can easily be incorporated in the feed and *L. casei* has known probiotic effects that facilitate the development of mucosal immunity. However, these types of vectors still have to be tested for their capacity to induce a good immune response, in particular against heterologous antigens, in broilers and whether they are able to provide protection against necrotic enteritis.

Another issue to be addressed when developing a vaccine against *C. perfringens*-induced enteric diseases is the choice of the antigens to be included in the vaccine. *C. perfringens*-induced diseases are the result of the toxins and enzymes that are produced and vaccination of chicks with *C. perfringens* supernatants provides protection against experimental necrotic enteritis [97, 105]. However, the protective capacity of the supernatants depends on the strain used for supernatant preparation, indicating that full protection might be determined by an effective combination of different bacterial immunogens [105]. In order to elucidate the optimal mixture of antigens to protect against necrotic enteritis, challenge trials are being performed mostly using parenteral vaccination schemes. Once the ideal combination of antigens is known, this will have to be adapted to oral delivery strategies. Several *C. perfringens* antigens have been evaluated as potential vaccine candidates. The tested antigens include both *C. perfringens* toxins (e.g. alpha toxin and the NetB toxin) and highly immunodominant proteins identified in post-infection serum from birds immune to necrotic enteritis [106]. In general, immunization studies of broilers with a single antigen all resulted in some level of protection against experimental necrotic enteritis. Remarkably, immunization with NetB toxin, which is essential to cause disease in broilers, does not afford higher levels of protection than vaccination with other toxins or proteins. However, when birds were vaccinated either via the parenteral or the oral route, with a combination of both NetB toxin and alpha toxin, higher levels of protection were obtained [107, 108]. In order to obtain full protection against *C. perfringens*-induced enteric diseases, not only antibodies that inhibit toxin activity might be needed; a combination of antigens targeting also bacterial proliferation, colonization and/or nutrient acquisition could be more efficient than either one of the individual approaches. Indeed, in a recent study disruption of the putative adhesin-encoding gene *cnaA* resulted in a reduced ability to colonize the chicken intestinal mucosa and to cause necrotic enteritis [109]. This strengthens the idea that vaccine antigens that target bacterial colonization might be indispensable to obtain a working vaccine against *C. perfringens*–induced enteric diseases. Additional vaccine targets might be enzymes that aid in breakdown of the host tissue and nutrient acquisition, such as, amongst others, mucinases, collagenases and hyaluronidases.

In contrast to the extensive efforts to develop a vaccine against necrotic enteritis in chickens, considerably less research has been directed to vaccination against necro-haemorrhagic enteritis in calves. The recent demonstration of the essential role of alpha toxin in necro-haemorrhagic enteritis and the proposition of a pathogenesis model will allow for the more targeted development of a vaccine [110, 111]. In calves as in chickens, protection against *C. perfringens*-induced necrosis can be obtained by antibodies against a mixture of toxins, at least in an experimental model for bovine necro-haemorrhagic enteritis [112]. Furthermore, antibodies against alpha toxin alone, which is essential to cause intestinal disease in calves, are not sufficient to provide the same level of protection as antibodies directed against a mixture of *C. perfringens* proteins, indicating that a mixture of different antigens will be needed to provide full protection [110]. In order to fully protect calves against *C. perfringens*-induced enteric diseases, antigens that target bacterial colonization and proliferation might be of equal importance as antigens targeting the toxin activities. Next, it has to be explored whether parenteral vaccination is sufficient to induce a protective immune response or if a combination of systemic and mucosal immunity is needed when not only the bacterial toxins but also bacterial colonization is targeted.

As administration of multiple parenteral doses of a vaccine to calves is more feasible than for chicken, it may be assumed that the development of a vaccine against necro-haemorrhagic enteritis is more straightforward and that *C. perfringens* supernatants can be used as a vaccine preparation. However, native toxins cannot be used as vaccine antigens due to safety issues. Inactivation of clostridial toxins is generally achieved by formaldehyde treatment, which risks residual formaldehyde in the vaccine preparation, incomplete inactivation of the toxins, and batch-to-batch variation. Moreover, formaldehyde inactivation can induce changes in the tertiary protein structures of relevant antigens and influence the immunogenicity of the vaccines. Indeed, vaccination of both chickens and calves with formaldehyde inactivated *C. perfringens* supernatants or toxins have resulted in a good antibody response, but these are unable to protect against intestinal disease [97, 112]. To overcome the need of chemically inactivating the *C. perfringens* toxins, current research focusses on the use of recombinant toxoids to develop a vaccine against *C. perfringens*-induced diseases. While this may be a good strategy to obtain a safe and protective vaccine on a laboratory scale, the production process is more laborious and time-consuming than production of conventional toxoids, especially because of the required purification steps [113]. Therefore, recent studies have explored the use of efficient low-cost alternatives, such as non-purified recombinant clostridial toxins and even recombinant bacterins, with success [114–116].

In summary of this section, considerable progress has recently been made in the development of efficacious vaccines against *C. perfringens*-induced enteric diseases. The main issue that hampers a breakthrough in this field is the identification of a defined combination of antigens that is able to provide full protection against disease. These antigens will most likely target both the bacterial toxins and the bacterial colonization and proliferation. For the broiler industry, once the ideal vaccine antigens have been identified, development of an oral vaccine is needed.

### Towards the development of new coccidiosis vaccines

Coccidiosis, an enteric disease cause by protozoan parasites of the genus *Eimeria*, remains a major economic and welfare concern for the poultry industry globally. Seven species (*Eimeria acervulina*, *E. brunetti*, *E. maxima*, *E. mitis*, *E. necatrix*, *E. praecox* and *E. tenella*) are known to infect chickens, and at least six others infect turkeys [117, 118]. The costs associated with coccidial disease are difficult to calculate, but have been estimated to exceed 3 billion US dollars for the chicken industry alone, worldwide [119]. Because coccidiosis is a predisposing factor for the occurrence of necrotic enteritis, the true economic burden is likely even higher. All *Eimeria* species can cause disease but the severity and clinical symptoms vary among species, and there is little or no cross-protection across species or some strains [120, 121].

#### Management of coccidiosis through anticoccidial drugs

Modern poultry production systems require effective control of coccidian parasites, typically through the routine use of anticoccidial drugs in feed or water. In the European Union, eleven different anticoccidial drugs are currently licensed and between 240 and 300 tonnes are sold for use in animals for markets such as the UK every year [122]. Anticoccidial drugs can be divided into two groups, synthetic or chemical anticoccidials and ionophores, which are products of fermentation [123]. In some countries such as the US, ionophores are classified as antibiotics, albeit with low human medical importance.

The ionophores currently dominate the anticoccidial drug market, largely because they provide incomplete protection, even against naïve field strains without any drug resistance. Low levels of parasites survive and induce protective immunity against the prevailing local parasite strains, without causing clinical disease [124]. Anticoccidial drugs provide an efficient means of controlling coccidial parasites and are highly cost-effective. However, drug resistance is widespread and increasing consumer concerns related to drug use in livestock production and residues in the food chain encourage the use of alternatives such as vaccination. Notably, because coccidiosis is a predisposing factor for necrotic enteritis and other secondary bacterial infections, efficient control of this parasite is important to minimize the use of medically important antibiotics, including those deemed critically important for human health, in poultry production.

#### Traditional live anticoccidial vaccines

The first anticoccidial vaccine was marketed in 1952^1^ [125]. It is a live parasite vaccine which includes multiple wild-type (i.e., non-attenuated) *Eimeria* species. Exposure to limited levels of such non-attenuated parasites permits the induction of a natural immune response in the chicken, resulting in protection against subsequent coccidial challenge. However, because protective immune responses against *Eimeria* are fully species specific, the inclusion of each individual target species is necessary if comprehensive protection is to be achieved, which results in relatively complex vaccine formulations. Such vaccines commonly include between three and eight parasite species or strains. The approach has been highly successful, although the lack of attenuation has been associated with reduced flock performance following vaccination and occasional clinical disease (reviewed elsewhere [126]).

In response to this limitation, a second generation of live *Eimeria* vaccines has been developed using attenuated parasite lines. For most of these vaccines, attenuation was achieved by selecting for so-called precocious strains, which typically exhibit reduced pathogenicity with fewer and/or smaller rounds of asexual replication. These attenuated strains retained their ability to immunize. The first live attenuated anticoccidial vaccine was launched in 1989,^2^ and several similar vaccines have been developed since using the same approach [126]. Non-attenuated and attenuated anticoccidial vaccines have become popular in the breeder and layer sectors, but are less widely used in the much larger broiler sector due to their relatively high cost compared to anticoccidial drugs and their limited availability. Because *Eimeria* cannot replicate effectively in vitro, the production of these live vaccines can only be achieved in *Eimeria*-free chickens and separate chickens have to be used for each species or strain to be included in a vaccine. Despite these production concerns billions of anticoccidial vaccine doses are sold every year, but more would be required to fully meet the growing demand.

#### Next generation anticoccidial vaccines

Efforts to improve on first and second generation live anticoccidial vaccines have included extensive attempts to identify antigens that are appropriate for use in subunit or recombinant vaccines. In addition, progress has been made on the preparation of novel adjuvants and some promising results have been obtained, although data on their use in poultry has so far remained fairly limited [127]. As an example, one vaccine^3^ is formulated from a crude mix of affinity purified *E. maxima* gametocyte antigens [128], although the levels of protection achieved have remained controversial and production of the vaccine still requires parasite amplification in chickens. Numerous studies have suggested that defined antigens such as apical membrane antigen 1, immune mapped protein 1, lactate dehydrogenase and SO7 are highly promising vaccine candidates (reviewed elsewhere [129]). Studies of *Eimeria* field populations have reported limited diversity in many of these antigens, indicating that recombinant vaccines for *Eimeria* may succeed even though antigenic diversity has undermined equivalent vaccines for related parasites such as *Plasmodium* [130, 131]. However, at present no recombinant anticoccidial vaccine is close to reaching the market.

One of the biggest remaining challenges is how to deliver the antigens in an affordable, effective, and, most importantly, scalable manner. A range of vectored expression/delivery systems have been suggested including Fowlpox virus (FWPV), HVT, *Salmonella* Typhimurium, yeasts such as *Saccharomyces cerevisiae* and the tobacco plant *Nicotiana tabacum*, with several showing promise [129]. Most recently, it has been suggested that *Eimeria* itself might function as an expression/delivery vector for vaccine antigens [132–134]. The ability to express and deliver anticoccidial vaccine antigens from multiple parasite species in a single transgenic line could provide an opportunity to streamline anticoccidial vaccine production from as many as eight lines to just one or two. Using an attenuated vector species such as *E. acervulina* can improve productive capacity enormously and reduce vaccine cost. The parasite vector may also provide some ability as an adjuvant and methods for on-farm delivery are well established [133].

In summary of this section on new coccidiosis vaccines, as pressure to reduce antibiotic drug use in livestock production increases it is clear that the demand for coccidial vaccines is stronger than ever. In the US, approximately 35–40% of broiler companies use programs that include vaccination to control coccidiosis [135]. This trend is primarily driven by demands to produce “no antibiotics ever” poultry products. However, it has also been shown that some coccidial vaccines provide an opportunity to replace drug-resistant field parasites in a poultry house with susceptible vaccine strains. While current European attenuated vaccines are limited by their lower reproductive potential, live vaccines do retain considerable unexplored potential. A better understanding of the underlying immune mechanisms through which these nontraditional approaches operate is needed to allow further progress. Ultimately, it is clear that novel vaccines must be cost-effective, compatible with high standards of animal welfare, scalable and easy to deliver.

## Autogenous vaccines to reduce the need for antibiotic use

Autogenous vaccines (AV) are also known as emergency, herd-specific or custom made vaccines. Although the legal basis and exact definition differs from country to country, AVs are used worldwide (e.g. EU, USA, Canada, Brazil, China, Indonesia, Australia, Egypt) and have a long history of use. The use of AVs for the control of fowl cholera has been well-documented [136, 137]. As a common definition, all AVs are made from inactivated bacterial or viral strains which were isolated from the same flock in which the vaccine is to be used. The use of AVs is only allowed if no licensed vaccine is available, or it is respectively ineffective or does not cover the current pathogen strains in the flock. The definition of a flock varies and may include integrated concepts of production chains in different places; to address the issue, the concept of an epidemiological link has recently been proposed by the Co-ordination Group for Mutual Recognition and Decentralised Procedures [138].

Licensed vaccines have advantages compared to AVs, including obligatory good manufacturing practice (GMP) production. Licensed vaccines are also produced in bigger batches with defined strains and a high level of quality which makes their efficacy and safety predictable. However, licensed vaccines are not available in all cases.

To generate AVs, selected bacterial or viral strains are usually combined with a proper adjuvant. Several viral or bacterial species can be used in a combination vaccine and different serotypes can also be combined in a polyvalent vaccine. The combination of inactivated viruses and bacteria is also an option. Bacterial AVs are accepted in all countries of the economic European area, whereas viral AVs are not allowed in 10 European countries including France, Denmark and Spain [138].

A critical role in the successful production and use of an AV falls to the isolation of vaccine strains. Therefore diagnostic samples must be carefully obtained, based on appropriate choices regarding which sick and untreated animals to select for sample collection, which necropsy material to select, and which cultivation conditions and strains to use after results from sero-, toxo- or virulence-typing. For that purpose several methods like PCR, MALDI-TOF MS, slide agglutination or DNA sequencing are available. Because of the fundamental importance of the strain choice for the production of an adequate AV, close collaboration between diagnostic laboratory and vaccine production is critical. Each production is custom-made and numerous adjuvants, viral and bacterial isolates, including serotypes, toxins and species, provide countless combinations. This underlines the importance of experience as the basis in the production of high quality AVs. The veterinarian also has obligations regarding diagnosis, ordering and responsibility for the administration of the vaccine.

A variety of bacterial components are often used in AVs. These include for poultry: *Bordetella* spp., *Campylobacter* spp., *Cl. perfringens*, *Enterococcus cecorum*, *Erysipelothrix rhusiopathiae*, *E. coli*, *Gallibacterium anatis*, *Mycoplasma spp.*, *Ornithobacterium rhinotracheale*, *Pasteurella multocida*, *Riemerella anatipestifer*; for swine: *Actinobacillus pleuropneumoniae*, *Bordetella* spp., *Brachyspira* spp., *Cl. perfringens*, *E. coli*, *H. parasuis*, *Mycoplasma* spp., *Pasteurella multocida*, *Strep. suis*, *Trueperella pyogenes*; for cattle: *Chlamydia* spp. *Cl. Perfringens*, *E. coli*, *Histophilus somni*, *Mannheimia haemolytica*, *Moraxella bovis*, *Mycoplasma* spp., *Pasteurella multocida*, *Salmonella enterica*, *Trueperella pyogenes*; and for fish: *Aeromonas* spp., *Photobacterium* spp., *Pseudomonas* spp., *Vibrio* spp., *Yersinia ruckeri.*

Depending on the animal species and age at vaccination different adjuvants can be used. As a standard adjuvant with good safety and efficacy, aluminium hydroxide is often used for production. Polymer and other gel-like adjuvants are also available for production in aqueous mixtures. Oily adjuvants, especially for water-in-oil emulsions, require a more sophisticated mixing procedure because of the need of a stable emulsion. Furthermore oily vaccines might pose safety concerns. However, these induce a promising long lasting immune response because of a depot effect. In the case of organic animal production use of plant oil might be an option in order to avoid unwanted hydrocarbons. The risk of adverse effects, which depend on the adjuvant-antigen combination, can be decreased by standardization of the protocols.

More data regarding the efficacy and safety of AVs in field studies should be collected because clinical safety and efficacy is not regulated. The need for this is reflected by numerous current publications about viral and bacterial AVs for poultry [139–142], bovine [143], swine [144] and fish [145]. Most results show that AVs can be a useful alternative to antibiotic use.

Only a few countries allow the use of live AVs [138]. The normally inactivated vaccines must be tested for sterility. In the EU this could be carried out by internal tests according to the Pharmacopoea [146]. Further steps in quality control include the inactivation test, endotoxin content or stability tests. Some producers offer GMP production, and GMP production is required in some countries such as Finland or Sweden [147]. In most countries GMP is only recommended. This example shows the vast differences in national legislation regarding the definition and interpretation of AVs. Because of worldwide circulation of animals and their pathogens a harmonization of manufacture, control and use of immunological veterinary medicinal products like AV is important, and the aim at the economic European area [138].

In summary, AVs are a valuable option in certain situations where commercial vaccines are either not available or expected to lack efficacy because of a mismatch between circulating and vaccine strains. The selection of adequate clinical isolates and vaccine formulations requires considerable expertise and the effective use of AVs depends on adequate manufacturing and appropriate veterinary oversight. Regulatory differences among countries create a highly fragmented legal landscape that would benefit from further harmonization.

## Conclusions

Vaccines are proven strategies for the prevention or control of infectious diseases in animal populations. Therefore, they are promising alternatives that can reduce the need to use antibiotics in food-producing animals and their direct mitigating impact on antibiotic consumption has been demonstrated in a number of studies, even though the relationship between antibiotic use and vaccination is not in all cases clear-cut. The ideal vaccine is safe, effective against a broad range of pathogens, and easily adapted to mass-application. At the same time, it is cheap to produce and use, easy to register across key jurisdictions, and generates durable protection, ideally after a single administration.

Existing vaccines still fall short of these ideals. In fact, many current vaccines have a number of shortcomings with regard to safety, efficacy and/or user-friendliness that limit their ability to replace antibiotic use. Overcoming these challenges will take close collaboration and innovative new approaches. Public–private partnerships represent one promising governing structure for assuring such close collaboration across public and private sectors. Investments in basic and applied research are equally needed to overcome these challenges, and research needs will have to be prioritized to ensure scarce resources will be preferentially dedicated to areas of greatest potential impact. Research to characterize and quantify the impact of vaccination on antibiotic use is equally needed.

Yet, some data demonstrating the ability of vaccines to reduce antibiotic consumption are already available. Similarly, key research breakthroughs and a number of highly promising vaccination approaches are already in development. These include new oral vaccines based on bacterial spores, live vectors, or new delivery strategies for inactivated oral vaccines; they also include new vaccination strategies in-ovo, combination vaccines that protect against multiple pathogens, the use of recent biotechnological advances, and comprehensive approaches to manage diseases caused by ubiquitous pathogens.

Therefore, further reductions in the need for antibiotic use through the use of new vaccines are all-but-certain, and investments in research and development of new vaccines will be vital for the sustained success of animal agricultural production around the world.

## Footnotes

1 Under the name CocciVac™.

2 Under the name Paracox™.

3 Under the name CoxAbic™.
